# Crystallographic fragment screen of the c-di-AMP-synthesizing enzyme CdaA from *Bacillus subtilis*

**DOI:** 10.1107/S2053230X24007039

**Published:** 2024-08-23

**Authors:** Tim Garbers, Piotr Neumann, Jan Wollenhaupt, Achim Dickmanns, Manfred S. Weiss, Ralf Ficner

**Affiliations:** a Department of Molecular Structural Biology, Institute of Microbiology and Genetics, GZMB Georg-August-University Göttingen Justus-von-Liebig-Weg 11 37073Göttingen Germany; b Macromolecular Crystallography Helmholtz-Zentrum Berlin 12489Berlin Germany; University of Leipzig, Germany

**Keywords:** CdaA, cyclic di-AMP, fragment screening, docking, novel antibiotics

## Abstract

Crystallographic fragment screening has become a pivotal technique in structure-based drug design, particularly for bacterial targets with a crucial role in infectious disease mechanisms. The enzyme CdaA, which synthesizes an essential second messenger cyclic di-AMP (c-di-AMP) in many pathogenic bacteria, has emerged as a promising candidate for the development of novel antibiotics. To identify crystals suitable for fragment screening, CdaA enzymes from *Streptococcus pneumoniae*, *Bacillus subtilis* and *Enterococcus faecium* were purified and crystallized. Crystals of *B. subtilis* CdaA, which diffracted to the highest resolution of 1.1 Å, were used to perform the screening of 96 fragments, yielding data sets with resolutions spanning from 1.08 to 1.87 Å. A total of 24 structural hits across eight different sites were identified. Four fragments bind to regions that are highly conserved among pathogenic bacteria, specifically the active site (three fragments) and the dimerization interface (one fragment). The coordinates of the three active-site fragments were used to perform an *in silico* drug-repurposing screen using the *OpenEye* suite and the DrugBank database. This screen identified tenofovir, an approved drug, that is predicted to interact with the ATP-binding region of CdaA. Its inhibitory potential against pathogenic *E. faecium* CdaA has been confirmed by ITC measurements. These findings not only demonstrate the feasibility of this approach for identifying lead compounds for the design of novel antibacterial agents, but also pave the way for further fragment-based lead-optimization efforts targeting CdaA.

## Abstract 2

The crystal structure determination of CdaA enzymes from *Streptococcus pneumoniae*, *Bacillus subtilis* and *Enterococcus faecium* is reported. Additionally, the structural results of a fragment screen of *B. subtilis* CdaA are presented, along with a subsequent *in silico* drug-repurposing screen conducted using the *OpenEye* suite.

## 1. Introduction

**Figure 1:**
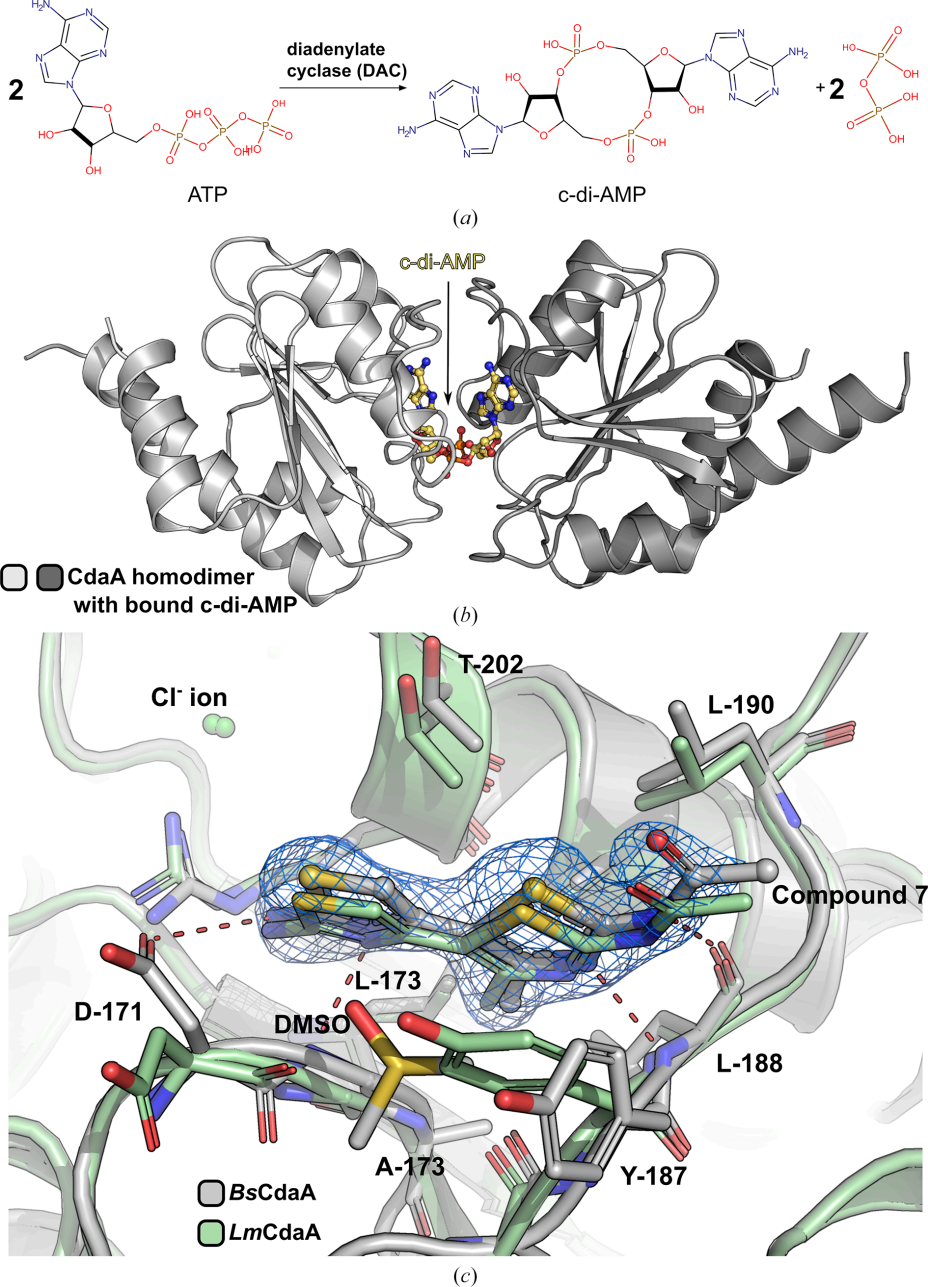
The crystal structure of the truncated form of *L. monocytogenes* CdaA, lacking the first 100 N-terminal residues and abbreviated Δ100. (*a*) The biochemical reaction catalyzed by diadenylate cyclase (DAC) involves the formation of cyclic di-AMP (c-di-AMP). (*b*) A cartoon representation depicting the catalytically active Δ100 CdaA dimer in its postcatalytic state with c-di-AMP bound in the active site (PDB entry 6hvl). (*c*) Superposition of *Bs*CdaA (gray) and *Lm*CdaA (pale green; PDB entry 8c4p) reveals an identical binding mode of the lead compound previously designed for *Lm*CdaA (Compound 7). The polder omit map (marine) is contoured at the +3σ level. The numbering corresponds to *Bs*CdaA.

Cyclic di-AMP (c-di-AMP) is a bacterial signaling dinucleotide that is predominantly found in Gram-positive bacteria. It plays a critical role in cell viability but becomes toxic when accumulated, as it is involved in various cellular processes, including DNA-integrity scanning, cell-wall metabolism and osmolyte homeostasis (Corrigan & Gründling, 2013 ▸; Commichau, Gibhardt *et al.*, 2018 ▸; Stülke & Krüger, 2020 ▸). Additionally, c-di-AMP regulates the bacterial stringent response, further underscoring its significance (Krüger *et al.*, 2021 ▸; Heidemann *et al.*, 2022 ▸). The synthesis of c-di-AMP (Fig. 1 ▸*a*) is mediated by enzymes possessing a diadenylate-cyclase (DAC) domain (Commichau, Heidemann *et al.*, 2018 ▸). These enzymes are categorized into five groups, CdaA, DisA, CdaS, CdaM and CdaZ, sharing the DAC domain but differing in additional domains or motifs (Sureka *et al.*, 2014 ▸). A notable feature of DAC proteins is their dimerization, which is essential for c-di-AMP synthesis (Fig. 1 ▸*b*). This process involves two DAC domain monomers, each binding one ATP molecule, aligning face to face to form a single reaction center (Müller *et al.*, 2015 ▸). The DAC domains of DisA, CdaS, and CdaA have been structurally characterized and share a similar structural architecture, characterized by seven parallel and antiparallel β-strands encircled by five α-helices (Fig. 1 ▸*b*; Rosenberg *et al.*, 2015 ▸). Enzymes facilitating c-di-AMP synthesis have been identified as potential targets for the development of new antibiotics due to their vital role in bacterial potassium and osmolyte homeostasis under normal growth conditions (Rosenberg *et al.*, 2015 ▸; Commichau, Heidemann *et al.*, 2018 ▸; Gundlach *et al.*, 2018 ▸). The deletion of genes encoding DAC enzymes has also been shown to increase bacterial sensitivity to β-lactam antibiotics, possibly due to compromised cell-wall stability (Dengler Haunreiter *et al.*, 2023 ▸). While certain *Bacillus* species possess up to three DAC protein variants, many pathogenic bacteria house only a single type, with CdaA being the sole DAC enzyme in pathogens such as *Staphylococcus aureus*, *Streptococcus pneumoniae*, *Listeria monocyto­genes* and *Enterococcus faecium*. This uniqueness positions CdaA as an ideal target for antibiotic development, meeting key criteria such as high conservation across bacterial species and a stringent correlation between gene deletion and reduced growth rates. Moreover, the absence of structural or functional homologs of the DAC domain in the human proteome could minimize the potential for drug side effects (Hughes & Karlén, 2014 ▸). Recently, we performed an *in silico* design of an *L. monocytogenes* CdaA inhibitor (Compound 7) that binds to *Lm*CdaA in the micromolar range with a dissociation constant (*K*_d_) approximately eight times lower than that of ATP (Neumann *et al.*, 2024 ▸). This bithiazole ring compound, which is distinct from adenosine, binds in the same position and thus serves as a promising lead compound for further optimization.

This report presents the crystal structure of the complex of *Bacillus subtilis* CdaA (*Bs*CdaA) and Compound 7, revealing the same binding mode as observed for *Lm*CdaA (PDB entry 8c4p; Fig. 1 ▸*c*), thereby providing evidence of high structural conservation of the CdaA ATP-binding site between bacterial species that are not closely related. Additionally, we report the first crystal structures of apo CdaA from *E. faecium* (*Ef*CdaA) and *S. pneumoniae* (*Sp*CdaA), as well as atomic resolution structures of *Bs*CdaA. Evaluation of the tested CdaA crystals from three different bacteria revealed that the *Bs*CdaA crystals were the most suitable for fragment screening. Our screening of the 96 compounds of the F2X-Entry Screen (Wollenhaupt *et al.*, 2020 ▸) yielded 24 crystal structures with bound fragments.

## 2. Materials and methods

### 2.1. Macromolecule production and ITC measurements

Standard molecular-biology procedures were employed to clone the CdaA enzymes from *S. pneumoniae*, *B. subtilis* and *E. faecium* (Table 1 ▸). Plasmid preparation was carried out using the Plasmid Prep Kit from Machery–Nagel following the manufacturer’s instructions. All proteins were expressed in *Escherichia coli* Rosetta (DE3) cells. For recombinant protein expression, 50 ml 2YT medium was supplemented with 50 µg ml^−1^ kanamycin and 34 µg ml^−1^ chloramphenicol and inoculated with several colonies of *E. coli* from an agar plate containing the desired plasmid. The culture was then incubated overnight at 37°C and 210 rev min^−1^. The expression medium was inoculated with 2 ml of the preculture and incubated at 37°C and 210 rev min^−1^ for 2 h. Subsequently, the temperature was reduced to 16°C and the cells were incubated for a further 72 h.

Expression cultures were harvested by centrifugation at 4800*g* at 4°C for 45 min. The supernatant was discarded and the cell pellet was resuspended in demineralized water. This suspension was centrifuged again at 4800*g* at 4°C for 30 min. The resulting cell pellet was flash-cooled in liquid nitrogen and stored at −20°C until further use. Cell lysis was performed by dissolving the pellet in a buffer consisting of 50 m*M* Tris–HCl pH 7.5, 500 m*M* NaCl, 20 m*M* imidazole, 10 m*M* MgCl_2_, a scoop tip of lysozyme and 0.1 U ml^−1^ DNase I. After 1 h of incubation, the cell suspension was passed seven times through a Microfluidizer 110S, where the cells underwent high-pressure treatment leading to cell-membrane rupture. The resulting cell lysate was clarified by centrifugation at 30 000*g* at 4°C for 45 min and manually filtered using a 0.45 µm cutoff filter. The clarified lysate then underwent a two-step chromatography purification protocol: Ni–NTA Sepharose affinity chromatography followed by size-exclusion chromatography (SEC). During the first purification step, a high-salt wash (1 *M* LiCl_2_) was employed to remove potential adenine derivatives bound to CdaA before the elution step. The eluates were incubated with PreScission protease [1:100(*w*:*w*)] to cleave off the N-terminal affinity tag and were subsequently treated with 10 m*M* EDTA to chelate divalent cations, which could otherwise induce sample heterogeneity. The final SEC purification was conducted in a buffer consisting of 10 m*M* Tris–HCl pH 7.5, 200 m*M* MgCl_2_ using a Superdex S200 column. The purity of the respective eluate fractions was confirmed by SDS–PAGE (17.5%, Coomassie Blue-stained), which displayed no additional bands.

ITC experiments were performed at 25°C and a stirring speed of 524 rev min^−1^ on a MicroCal VP-ITC microcalorimeter (MicroCal). Measurements were carried out with 75 µ*M* CdaA in the sample cell and 1–2 m*M* of the analyzed ligand in the titration syringe (tenofovir disoproxil, ATP). Both the protein and ligands were dissolved in the same buffer composed of 20 m*M* Tris–HCl pH 7.5, 300 m*M* NaCl. The buffer was supplemented with 5% DMSO and 10 m*M* MgCl_2_. One control experiment was carried out: titrant into buffer. Data were analyzed using the *MicroCal PEAQ-ITC Analysis Software* version 1.41 (Malvern Panalytical) employing the single control method (subtraction of titrant into buffer experiment). For all performed experiments, the data sets were fitted with a 1:1 binding model and yielded an assessment of the following thermodynamic parameters: dissociation constant (*K*_d_) and enthalpy of interaction Δ*H*.

### 2.2. Crystallization of CdaA enzymes and fragment-screening campaign with *Bs*CdaA crystals

Crystallization experiments were conducted for all three CdaA enzymes (*Sp*CdaA from *S. pneumoniae*, *Ef*CdaA from *E. faecium* and *Bs*CdaA from *Bacillus subtilis*; Table 2 ▸) at 293 K using a Mosquito pipetting robot (SPT Labtech) with MRC 96-well 3-lens plates (SWISSCI 3 Lens Crystallization Midi Plate UVP). Protein concentrations ranged from 4 to 6 mg ml^−1^ (Table 2 ▸) with a droplet size of 500 nl and protein:reservoir ratios of 1:1 and 2:1. Upon identifying initial crystallization conditions, fine screening was carried out using 24-well sitting-drop vapor-diffusion plates with a reservoir volume of 500 µl, a drop volume ranging between 1.5 and 4 µl, and varying protein:reservoir ratios. The concentrations of single reservoir components and the protein were adjusted accordingly. Additionally, DMSO was added to some crystallization conditions to enhance the solubility of fragments in subsequent soaking experiments. CdaA from *B. subtilis* and *S. pneumoniae* yielded crystals under low-salt conditions using PEG 400 or PEG 3350 as precipitants, whereas *Ef*CdaA crystals grew in conditions containing 1.5 *M* lithium sulfate. The crystal diffraction limits varied notably, with *Ef*CdaA diffracting to 2.45 Å resolution, *Sp*CdaA to 1.64 Å resolution and *Bs*CdaA to 1.1 Å resolution, prompting the selection of the *Bs*CdaA crystals for subsequent fragment screening due to their high reproducibility, superior diffraction properties and low-salt crystallization conditions. It should be noted that the *Sp*CdaA crystals were difficult to reproduce and rarely diffracted to a resolution better than 2.0 Å. This could potentially be related to the larger number of protein molecules that occupy the asymmetric unit (six for *Sp*CdaA versus two for *Bs*CdaA and *Ef*CdaA) and the elevated solvent content of 56.3% compared with 47.3% for *Bs*CdaA crystals.

To enhance fragment screening, we optimized the crystallization conditions of *Bs*CdaA in its apo state, focusing on crystal size and reproducibility. Optimization trials were performed in the aforementioned crystallization plates (Mosquito pipetting robot), aiming to produce at least 300 crystals. The original condition, with 30%(*v*/*v*) PEG 400 content, acted as a cryoprotectant. Therefore, we varied the pH and the salt and protein concentrations. Maintaining a concentration of 6 mg ml^−1^*Bs*CdaA with 30%(*v*/*v*) PEG 400, 100 m*M* HEPES (pH 7.5) and 100 m*M* MgCl_2_ consistently yielded crystals of approximately 200 × 100 × 50 µm in size, facilitating easy crystal transfer and harvesting processes. Different DMSO concentrations (5%, 10%, 15%, 20% and 25%) were tested for their effects on crystal size. The 20% DMSO concentration yielded the largest crystals (up to 500 × 100 × 50 µm) without skin formation, unlike the 25% DMSO condition. The diffraction properties, tested across five crystals per DMSO concentration, consistently ranged between 1.4 and 1.5 Å resolution, leading to the incorporation of 20% DMSO in subsequent crystallization experiments.

Crystallographic fragment screening of *Bs*CdaA utilized the F2X-Entry Screen (Wollenhaupt *et al.*, 2020 ▸) comprising 96 fragments. Each fragment was present in a dried form in two lenses of the respective well of an MRC 96-well 3-lens low-profile crystallization plate (Wollenhaupt *et al.*, 2020 ▸). The reservoir of each well was filled with 40 µl crystallization buffer. Solubilization of the dried fragments was achieved by pipetting 0.4 µl of the crystallization reservoir supplemented with 20% DMSO into one of the two lenses, resulting in a nominal fragment concentration of 100 m*M*. Four to five crystals were transferred into each drop. To reduce evaporation during the transfer process, a novel evaporation-protecting device was used (Barthel *et al.*, 2021 ▸). The plate was sealed and the crystals were incubated at 20°C overnight. The same approach was used to perform soaking experiments on *Bs*CdaA crystals with Compound 7. Prior to this, the compound was dissolved in DMSO, pipetted into two lenses and dried out.

### 2.3. Data collection and processing

Diffraction data were collected from crystals at 100 K. Initially, crystals were tested on an in-house MicroMax-007 rotating-anode X-ray generator (Rigaku) equipped with a MAR image-plate detector. Crystallographic data sets were collected on beamlines P13 and P14 at DESY Hamburg and beamline 14.1 at BESSY II in Berlin. Typically, a full 360° rotation of a crystal was collected using an incremental step of 0.1°. However, to reduce the total data-collection time for soaked crystals (F2X-Entry Screen) only 270° of data were collected with an oscillation range of 0.15°. Diffraction images were processed using the *XDS* program package (Kabsch, 2014 ▸), either using a homemade pipeline (Neumann *et al.*, 2024 ▸) or, in the case of the fragment-screening campaign, via *FragMAXapp*. All data sets were analyzed using the *XDSSTAT* program to evaluate the amount of radiation damage. Notably, no radiation damage was observed in any of the collected data sets. Diffraction images for the *Bs*CdaA–Compound 7 complex were collected using an in-house rotating-anode generator (oscillation range 0.5°, 546 images) and were processed with *XDS*. Data-collection and processing statistics are summarized in Table 3 ▸.

### 2.4. Structure solution, refinement and fragment-based *in silico* drug repurposing using the *OpenEye* suite

The structures of *Bs*CdaA, *Sp*CdaA and *Ef*CdaA were determined using molecular replacement with *DIMPLE* and *Phaser* (Wojdyr *et al.*, 2013 ▸; McCoy *et al.*, 2007 ▸) utilizing previously published CdaA structures as search models (PDB entries 6huw, 6gyw and 7l8n, respectively). For *Sp*CdaA and *Ef*CdaA, the sequences of the employed search models were adjusted using *CHAINSAW* (Stein, 2008 ▸) prior to molecular-replacement searches. The structural models were refined using a customized self-written refinement pipeline utilizing *CCP*4 (Winn *et al.*, 2011 ▸) and *Phenix* (Liebschner *et al.*, 2019 ▸). Atomic models were manually adjusted in *Coot* (Emsley *et al.*, 2010 ▸). Refinement statistics are summarized in Table 4 ▸. Structural models derived from the fragment-screening campaign were refined using a pipeline embedded in *FragMAXapp* utilizing *DIMPLE* (*CCP*4) and *phenix.refine* (*Phenix*). The apo structure of *Bs*CdaA (PDB entry 6huw) served as the initial model. Data analysis facilitated *FragMAXapp* (Lima *et al.*, 2021 ▸) and *PanDDA* (Pearce *et al.*, 2017 ▸) in detecting fragment hits. From 203 crystals soaked with 96 different fragments, 201 data sets were collected, revealing a hit rate of 33%, with 24 distinct fragments interacting with *Bs*CdaA across eight sites (Supplementary Table S1). Atomic models with identified fragments underwent further manual rebuilding in *Coot* alternated with reciprocal-space and real-space refinement cycles using the aforementioned self-written pipeline (a tcsh script is provided as supporting information). The corresponding structural models with identified fragment molecules were deposited: A09, PDB entry 8ogn; A12, PDB entry 8ogo; B03, PDB entry 8ogp; B06, PDB entry 8ogq; B07, PDB entry 8ogr; B08, PDB entry 8ogs; C04, PDB entry 8ogt; C07, PDB entry 8ogu; C08, PDB entry 8ogv; D02, PDB entry 8ogw; D04, PDB entry 8ogy; D06, PDB entry 8ogz; D08, PDB entry 8oh0; E04, PDB entry 8oh1; E08, PDB entry 8ohb; E12, PDB entry 8ohc; F03, PDB entry 8ohe; F04, PDB entry 8ohf; F09, PDB entry 8ohg; G05, PDB entry 8ohh; G08, PDB entry 8ohj; H01, PDB entry 8ohk; H09, PDB entry 8ohl; H11, PDB entry 8oho; Compound 7, PDB entry 9g0g. All reported structural models and experimental data are accessible via the Protein Data Bank. Data-collection and refinement statistics, as calculated with the *phenix.table1* program, are summarized in Supplementary Table S2. All figures were prepared using the open-source version of *PyMOL* (version 2.6; Schrödinger). The sequence-conservation score was calculated using the *ConSurf* server (Yariv *et al.*, 2023 ▸) with default settings. The scores were grouped into nine conservation grades ranging from 1 to 9. Grade 1 includes the most rapidly evolving positions, grade 5 includes positions with intermediate rates of evolution and grade 9 includes the most evolutionarily conserved positions.

Based on the three crystal structures with small-molecular fragments bound in the CdaA active site (A09, B08 and D02, Fig. 3), we initiated an *in silico* drug-repurposing search. The primary objective was to identify an existing drug or drug candidate capable of mimicking the binding positions of the three identified fragment molecules and potentially binding to the ATP-binding site of CdaA, even if it was not originally developed for this purpose. The structure-based *in silico* drug repurposing was conducted using the *OpenEye* suite (https://www.eyesopen.com/; academic license) with the DrugBank library (https://go.drugbank.com/; academic license). The DrugBank data set (released on 14 March 2024) underwent preprocessing with the *OMEGA* program (version 5.0.0.3; https://docs.eyesopen.com/applications/omega/introduction.html).*OMEGA* generated up to 600 3D conformations of each molecule in the DrugBank library. These conformers were then utilized as input for *ROCS* (version 3.6.1.3; https://docs.eyesopen.com/applications/rocs/index.html). The ligand-based lead-discovery software *ROCS* identifies potentially active leads by comparing molecules based on shape and chemical features defined by the user, such as positions of hydrogen-bond donors, acceptors, aromatic ring structures and cation–π stacking possibility. Four *ROCS* shape queries were prepared based on the bound fragment molecules (Supplementary Tables S3*a* and S3*b*; the sheet entitled ROCS QUERIES). For each query, three different *ROCS* searches were performed using an ensemble of 3D conformers generated by *OMEGA*. These searches utilized three different *ROCS* scoring functions: TanimotoCombo, RefTverskyCombo and FitTverskyCombo (for details, please refer to https://docs.eyesopen.com/applications/rocs/theory/shape_theory.html). The resulting drug and drug-candidate conformers (200 for each of 12 runs) identified by *ROCS* as the best-fitting potential lead compounds underwent docking analysis.

Molecular docking was conducted using the *HYBRID*program (version 4.3.0.3; https://docs.eyesopen.com/applications/oedocking/hybrid/hybrid.html) from the *OpenEye* suite, employing structural models of *Bs*CdaA molecules bound with fragments as targets. Drug and drug-candidate molecules (ligands) identified by *ROCS* and subsequently docked by *HYBRID* underwent a second round of docking. The predicted ligand–protein pairs from *HYBRID* were then subjected to docking with the *Gnina* program (McNutt *et al.*, 2021 ▸), which employs an ensemble of convolutional neural networks (CNNs) for scoring. The *Gnina* CNN score offers insight into the likelihood of a pose being within 2 Å of the true binding pose, serving as a quality indicator for ligand conformation. During cross-docking, a CNN score above 0.8 indicates a minimum 56% probability of achieving a 2 Å r.m.s.d., which increases to 79% with redocking (McNutt *et al.*, 2021 ▸). *Gnina* docking experiments utilized two commonly used scoring functions, Vina and Vinardo, with standard settings except for the exhaustiveness parameter, which was increased from its default of 8 to 64 to enhance search efficiency. The rationale behind the second docking round was to refine potential lead candidates by selecting those that perform well in both the *HYBRID* and *Gnina* approaches. Docking results were filtered based on criteria including a *HYBRID* Chemgausscore4 between −3.2 and −7.5, a *Gnina* CNN score of >0.8 and an r.m.s.d. between *HYBRID* and *Gnina* poses initially set to below 4 Å and later adjusted to 6 Å based on manual inspection. Ligands were retained if any of the nine binding poses predicted by *Gnina* fell within the 6 Å r.m.s.d. threshold of the binding pose proposed by *HYBRID*. All docking-related results, comprising the pool of 53 identified ligands that fulfilled the aforementioned criteria, are summarized in Supplementary Tables S3(*a*) and S3(*b*). These tables include 2D ligand representations, SMILES strings, molecular masses, DrugBank IDs, drug group classifications and more. Additionally, they contain screenshots of the employed ROCS QUERIES, as well as *PyMOL*-generated figures illustrating all 53 predicted ligand-binding modes plus those of tenofovir disoproxil (15A) and tenofovir alafenamide (15B), saved in individual sheets of the *MS Excel* file. Supplementary Table S3(*a*) contains the first half of the figures and Supplementary Table S3(*b*) contains the other half. Each figure depicts both the *HYBRID* and *Gnina* predicted binding modes for comprehensive comparison (not in the case of tenofovir derivatives).

## 3. Results and discussion

**Figure 2:**
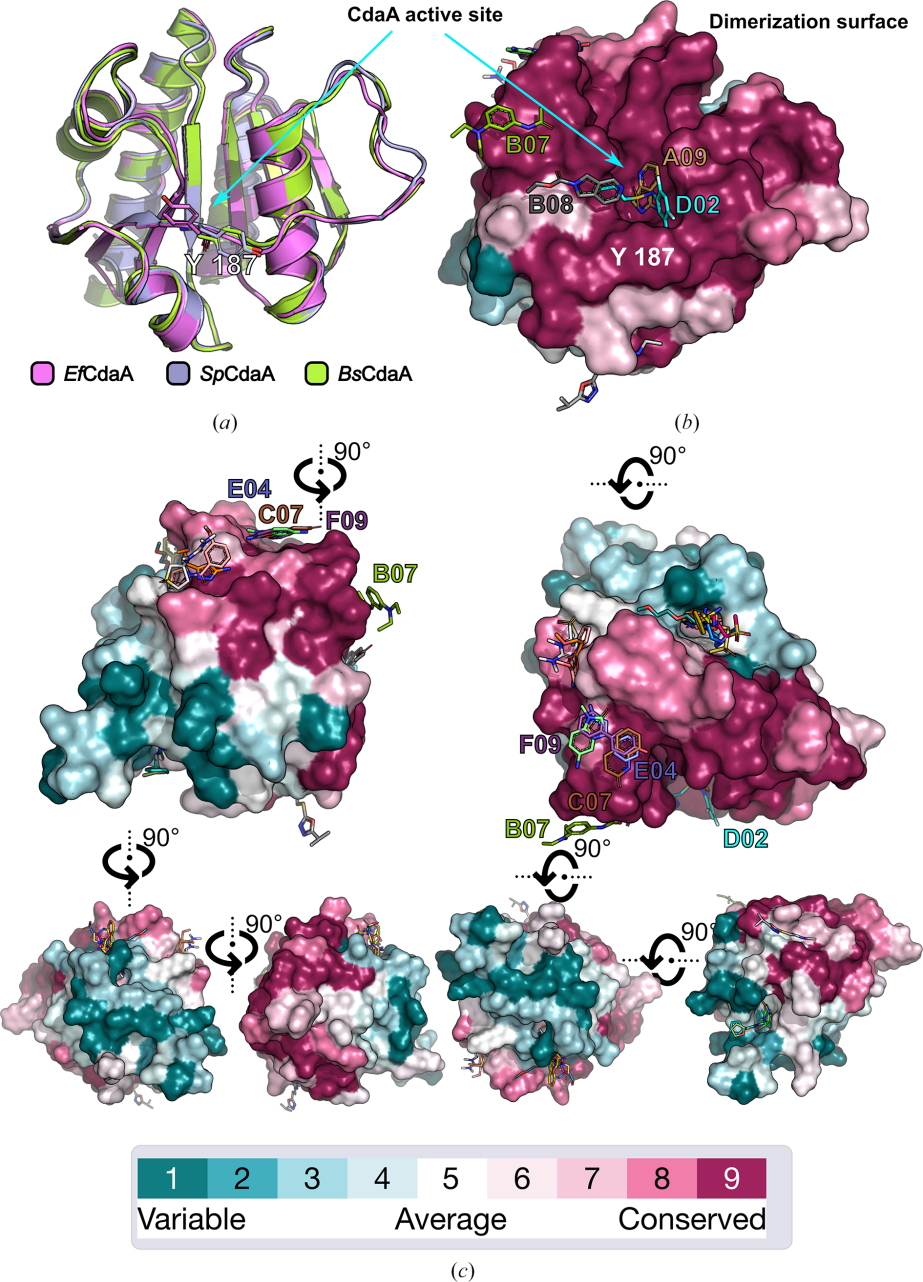
The crystal structures of the discussed N-terminally truncated bacterial CdaAs are presented. (*a*) Superposition of *S. pneumoniae* (violet), *B. subtilis* (lime) and *E. faecium* (light blue) CdaA monomers reveals high structural similarity between the reported structures. A conserved tyrosine residue important for catalytic activity is depicted in stick representation. (*b*) Surface representation of the *Bs*CdaA monomer colored by sequence-conservation score. The orientation corresponds to that in (*a*). Fragment molecules are depicted as sticks, and those bound to sequence-conserved areas are labeled. (*c*) Different orientations of the *Bs*CdaA monomer depicted in (*b*).

Crystallographic fragment screening requires a significant quantity of well diffracting crystals of consistent quality. Previous studies have reported that crystals of CdaA from *L. monocytogenes* in its apo form achieved resolutions of 1.45–2.2 Å (Heidemann *et al.*, 2019 ▸; Neumann *et al.*, 2024 ▸), but these crystals grew under high-salt concentrations of up to 4.5 *M* ammonium sulfate, which could potentially alter the protein–ligand interactions and reduce fragment solubility. A fragment screen conducted on *Lm*CdaA crystals yielded only eight bound fragments (Neumann *et al.*, 2024 ▸). To address these issues, we aimed to grow CdaA crystals under low-salt conditions. However, suitable crystallization conditions for unliganded *Lm*CdaA could not be identified. Therefore, structurally related CdaA enzymes from *S. pneumoniae*, *B. subtilis* and *E. faecium* were cloned, overexpressed in *E. coli*, purified, crystallized and evaluated for diffraction quality (Tables 1 ▸, 2 ▸ and 3 ▸). The obtained atomic models exhibit a high degree of structural similarity (Fig. 2 ▸), as shown by the calculated root-mean-square deviation (r.m.s.d.) values. Model comparisons reveal deviations of 0.496 Å for 132 C^α^ positions between the best-diffracting *Bs*CdaA (colored lime) and *Ef*CdaA (colored violet) crystals and of 0.392 Å for 109 C^α^ positions between *Bs*CdaA and *Sp*CdaA (colored light blue) (Fig. 2 ▸). Despite the observed structural similarity, the sequence conservation varies considerably across the sequence, with the highest conservation at the ATP-binding site and dimerization surface (Fig. 2 ▸). With the goal of developing an inhibitory compound against CdaA from different pathogenic organisms, we decided *a priori* to focus primarily on fragment hits that bind to regions of high sequence conservation: the ATP-binding site and dimerization surface (Fig. 2 ▸, Supplementary Table S1). The reported structure of *Bs*CdaA in complex with an inhibitor (Compound 7) originally developed for *Lm*CdaA, which reveals an identical ligand-binding mode, provides structural evidence supporting the efficacy of this approach. Consequently, we selected *B. subtilis* CdaA for the fragment-screening campaign due to the excellent diffraction properties, high reproducibility and quality of *Bs*CdaA crystals. Notably, these crystals tolerated up to 20% DMSO in the reservoir solution, which facilitates soaking experiments. A crystallographic fragment screen of *Bs*CdaA utilizing the F2X-Entry Screen resulted in a 33% hit rate, identifying 24 distinct fragments interacting with *Bs*CdaA across eight sites (Supplementary Tables S1 and S2). Analysis of these hits narrowed the selection down to a total of four different fragments that can serve as starting points for the development of inhibitory lead compounds.

**Figure 3:**
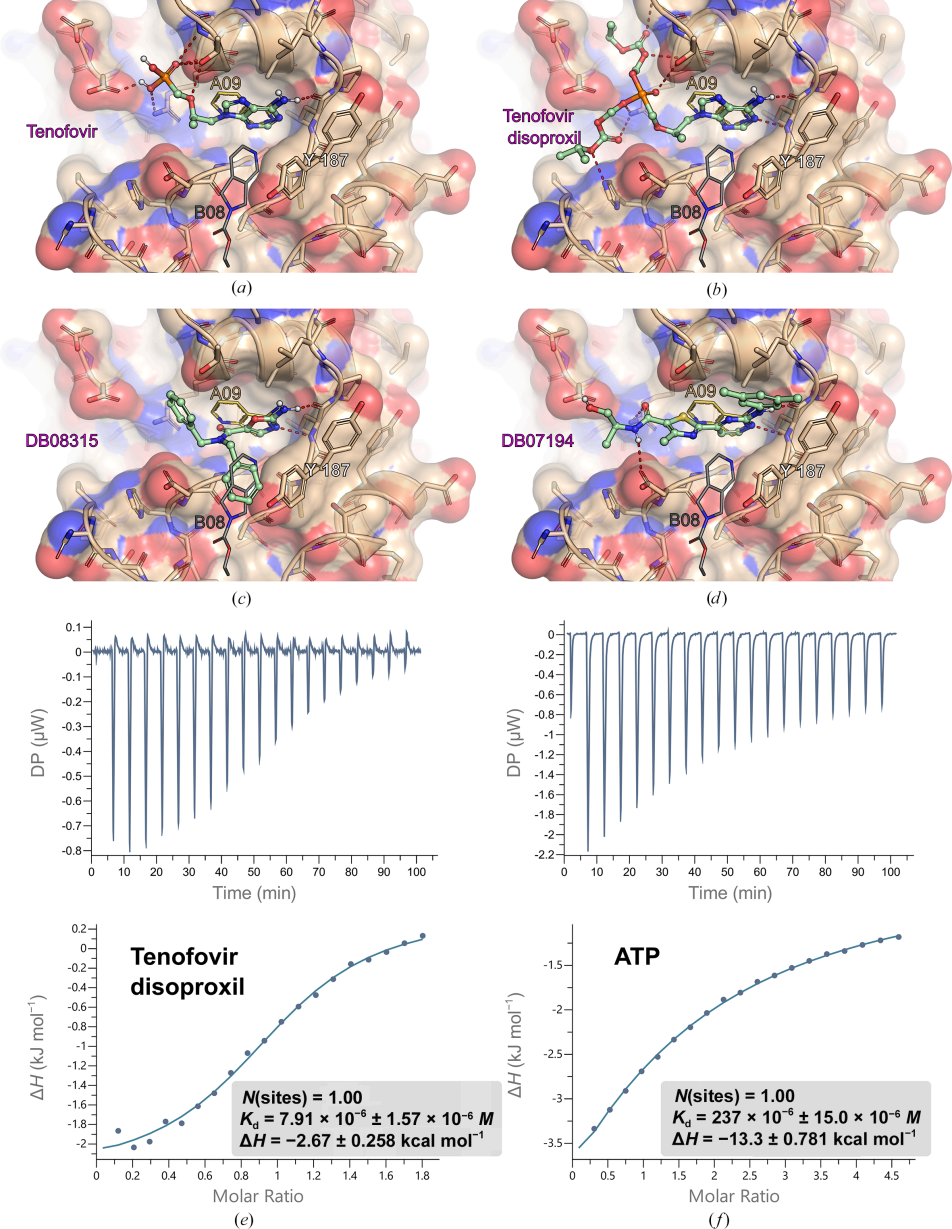
Fragment-based *in silico* drug repurposing was conducted using the *OpenEye* suite and *Gnina*. *Bs*CdaA is depicted in stick representation (with carbon in wheat, oxygen in red and nitrogen in blue) with a transparent atom-colored surface. Tyr187, which is crucial for catalysis, is highlighted. Two bound fragments (thin stick models, labeled A09 and B08) are shown, while the third fragment (D02) was omitted for clarity. Identified compounds are depicted in ball-and-stick representation (carbon in pale green) and labeled either with their commercial name (approved drugs) or with the DrugBank ID (experimental drugs). (*a*) Tenofovir, (*b*) tenofovir disoproxil, (*c*) DB08315, (*d*) DB07194; (*e*) and (*f*) present thermodynamic analysis of tenofovir disoproxil and ATP binding to *Ef*CdaA using isothermal titration calorimetry (ITC). The raw data thermogram (top) and the integrated heats (bottom) are presented. The data were fitted with a 1:1 binding model and yielded the dissociation constant (*K*_d_) and the enthalpy of interaction Δ*H*.

These fragments (A09, B08, D02 and B07), which bind to highly conserved areas of CdaA (Fig. 2 ▸*b*), particularly at the site where the adenine moiety of ATP binds, offer potential as chemical groups for the design of adenine derivatives or modifying the recently published *in silico*-designed *Lm*CdaA inhibitor (Neumann *et al.*, 2024 ▸). This approach aims to develop a more CdaA-specific inhibitor, distinct from adenosine. Building on this, we conducted an *in silico* drug-repurposing screen using the *OpenEye* suite (with a free academic license) and the DrugBank database (also with a free academic license) to explore potential lead compounds for novel CdaA inhibitors (Supplementary Tables S3*a* and S3*b*). This also allowed us to investigate the complementarity at the molecular level of chemical groups building known drug molecules and the ATP-binding site of CdaA. These functional groups could serve as hints for alteration strategies to increase the water solubility of a lead compound designed by us (Compound 7), such as the addition of a disoproxil moiety. Among the 53 identified compounds, one is approved, 18 are investigational and 27 are experimental drugs. The most promising candidates (Fig. 3 ▸) were selected based on the highest CNN scores (>0.9) and the lowest similarity to the substrate molecule. Their predicted binding modes mirror crucial interactions observed in previously solved CdaA structures with ATP and c-di-AMP (Heidemann *et al.*, 2019 ▸; Neumann *et al.*, 2024 ▸). The top-scoring drug identified *in silico* was tenofovir, an acyclic nucleotide diester analog of adenosine monophosphate used to treat HIV and hepatitis B infections (Fig. 3 ▸*a*). Due to its low oral bioavailability, it is commercially available in the form of tenofovir disoproxil and tenofovir alafenamide. While both tenofovir derivatives were docked to CdaA using *Gnina*, only tenofovir disoproxil scored significantly highly (CNN score 0.92; Fig. 3 ▸*b*, Supplementary Table S3*a*; compounds labeled 15A and 15B) and its predicted binding mode resembled that of tenofovir and the adenine moiety known from the *Lm*CdaA–ATP (PDB entry 8c4o) and *Lm*CdaA–AMP (PDB entry 8c4n) complexes. Despite the fact that tenofovir disoproxil has been reported to lack antibacterial activity (Rubio-Garcia *et al.*, 2024 ▸), it can still be considered as a lead compound that likely cannot cross the bacterial cell membrane. Remarkably, ITC measurements of tenofovir disoproxil showed inhibitory potential on *Ef*CdaA, revealing a *K*_d_ (equilibrium dissociation constant) about 30 times lower than that of the natural substrate ATP (Fig. 3 ▸). This compound would certainly require further modifications, such as, for example, the incorporation of a siderophore moiety, to increase its uptake by bacteria. Additionally, two experimental drugs were identified: 2-amino-*N*,*N*-bis(phenyl­methyl)-1,3-oxazole-5-carboxamide (DB08315; Fig. 3 ▸*c*) and 2-{2-[(3,5-dimethylphenyl)amino]pyrimidin-4-yl}-*N*-[(1*S*)-2-hydroxy-1-methylethyl]-4-methyl-1,3-thiazole-5-carboxamide (DB07194; Fig. 3 ▸*d*). Alternatively, the three identified fragments (A09, B08 and D02) could be used to identify a new chemical scaffold that is distinct from adenosine but would bind in its position. This is of particular interest because adenosine is an important intermediary metabolite, acting as a building block for nucleic acids and a key component of biological energy storage. Additionally, adenosine functions as a signaling molecule through the activation of four distinct adenosine receptors: A1, A2A, A2B and A3. These receptors are widely expressed and have been implicated in numerous physiological and pathological processes (Chen *et al.*, 2013 ▸). An inhibitor that mistakenly targets these receptors could cause severe side effects. Another challenging alternative would be to design a molecule that could link fragments A09, B08, D02 and B07 to simultaneously block the active site and prevent the formation of the catalytically active dimer.

In conclusion, we have demonstrated that a structurally related enzyme from a nonpathogenic bacterium can be used to identify crystallization conditions that yield crystals suitable for a crystallographic fragment-screening campaign. This approach allows us to avoid reservoir solutions with high salt concentrations, potentially enabling the identification of a larger pool of fragment molecules that bind to sequence-conserved areas of CdaA. The identified fragments enhance our molecular toolkit against DAC enzymes and highlight the potential of targeting these enzymes for antibiotic development. Additionally, by leveraging these fragments, we conducted an *in silico* drug-repurposing search, which led to the identification of tenofovir disoproxil, an approved drug, as a potential lead candidate for the development of a CdaA inhibitor.

## Supplementary Material

PDB reference: *Streptococcus pneumoniae* CdaA, 8ofh

PDB reference: *Enterococcus faecium* CdaA, 8ofo

PDB reference: *Bacillus subtilis* CdaA, 8ogm

PDB reference: complex with fragment A09, 8ogn

PDB reference: complex with fragment A12, 8ogo

PDB reference: complex with fragment B03, 8ogp

PDB reference: complex with fragment B06, 8ogq

PDB reference: complex with fragment B07, 8ogr

PDB reference: complex with fragment B08, 8ogs

PDB reference: complex with fragment C04, 8ogt

PDB reference: complex with fragment C07, 8ogu

PDB reference: complex with fragment C08, 8ogv

PDB reference: complex with fragment D02, 8ogw

PDB reference: complex with fragment D04, 8ogy

PDB reference: complex with fragment D06, 8ogz

PDB reference: complex with fragment D08, 8oh0

PDB reference: complex with fragment E04, 8oh1

PDB reference: complex with fragment E08, 8ohb

PDB reference: complex with fragment E12, 8ohc

PDB reference: complex with fragment F03, 8ohe

PDB reference: complex with fragment F04, 8ohf

PDB reference: complex with fragment F09, 8ohg

PDB reference: complex with fragment G05, 8ohh

PDB reference: complex with fragment G08, 8ohj

PDB reference: complex with fragment H01, 8ohk

PDB reference: complex with fragment H09, 8ohl

PDB reference: complex with fragment H11, 8oho

PDB reference: complex with Compound 7, 9g0g

Table S1. F2X-Entry Screen Fragment Hit List. DOI: 10.1107/S2053230X24007039/no5208sup1.xlsx

Table S2. Data collection and refinement statistics (crystal structures related to fragment screen). DOI: 10.1107/S2053230X24007039/no5208sup2.xlsx

Table S3a. In silico drug repurposing using the OpenEye suite and Gnina. For the first 27 compounds from the DrugBank repository, the detailed binding interactions are shown as individual figures. The remaining figures are included in Table S3b. DOI: 10.1107/S2053230X24007039/no5208sup3.xlsx

Table S3B. In silico drug repurposing using the OpenEye suite and Gnina. The remaining figures that are not included in Table S3a. DOI: 10.1107/S2053230X24007039/no5208sup4.xlsx

Tcsh script used for automatic refinement of deposited structures (the self-written refinement pipeline). DOI: 10.1107/S2053230X24007039/no5208sup5.txt

## Figures and Tables

**Table 1:** Macromolecule-production information

Source organism	*S. pneumoniae*	*E. faecium*	*B. subtilis*
DNA source	Codon-optimized synthetic DNA	Codon-optimized synthetic DNA	*B. subtilis*
Expression vector	pET-28a	pET-28a	pET-28a
Plasmid-construction method	Restriction–ligation	Restriction–ligation	Restriction–ligation
Forward primer	CTAGCTAGCCTGGAAGTGCTGTTTCAGGGTCCGGCCGAAGAACAGATGATTCGTGC	CTAGCTAGCCTGGAAGTGCTGTTTCAGGGTCCGAGCGAACAGCAGGAAGATGAAAAAATG	CTAGCTAGCCTGGAAGTGCTGTTTCAGGGTCCGACGATTGAGGCCATTACAAAAGC
Reverse primer	CGGGATCCTTAACCAAACCAATGTTCTGCCAGCAG	CGGGATCCTTAAATCAGTTCACGACGCAGAATGGCC	GCGGGATCCTTAAAACTCGGCTTCAAGCATTTCTTTCAGC
Expression host	*E. coli* Rosetta (DE3)	*E. coli* Rosetta (DE3)	*E. coli* Rosetta (DE3)
Complete amino-acid sequence of the construct produced	GSHMASMNAPISAEEQMIRAFVKSVEYMSPRKIGALVAIQRVRTLQEYISTGIPLDAKISAELLINIFIPNTPLHDGAVIIKEERIAVTSAYLPLTKNTGISKEFGTRHRAAIGLSEVSDALTFVVSEETGGISITYNGRFKHNLTLDEFETELREILLPKEEVGLSFKERLLGGWKHEKK	GPQQEDEKMILSFDKAIQYMSKRKIGALITIERHTGLDEYIETGIALDADITGELLINIFIPNTPLHDGAVIVKEGKIAVASAYLPLSESMLIPKEFGTRHRAAVGISEVSDAITIVVSEETGDVSITLDNELMAGLSQQEYLAILRRELI	GPTPVEEAQQKTIEAITKAINYMAKRRIGALLTIERDTGMGDYIETGIPLNAKVSSELLINIFIPNTPLHDGAVIMKNNEIAAAACYLPLSESPFISKELGTRHRAAVGISEVTDSLTIIVSEETGGVSVAKNGDLHRELTEEALKEMLEAEFK

**Table 2:** Crystallization

	*Sp*CdaA	*Ef*CdaA	*Bs*CdaA
Method	Vapor diffusion	Vapor diffusion	Vapor diffusion
Plate type	Sitting drop	Sitting drop	Sitting drop
Temperature (K)	293	293	293
Protein concentration (mg ml^−1^)	6	5.5	4
Buffer composition of protein solution	10 m*M* Tris–HCl pH 7.5, 200 m*M* MgCl_2_	10 m*M* Tris–HCl pH 7.5, 200 m*M* MgCl_2_	10 m*M* Tris–HCl pH 7.5, 200 m*M* MgCl_2_
Composition of reservoir solution	100 m*M* bis-Tris propane pH 7.5, 200 m*M* KSCN, 15%(*w*/*v*) PEG 3350	50 m*M* Tris–HCl pH 8.5, 5 m*M* MgCl_2_, 1.5 *M* Li_2_SO_4_, 5%(*v*/*v*) glycerol	100 m*M* HEPES pH 8.0, 200 m*M* MgCl_2_, 30%(*v*/*v*) PEG 400
Volume and ratio of drop	0.5 µl, 1:1 ratio	0.5 µl, 1:1 ratio	0.5 µl, 1:1 ratio
Volume of reservoir (µl)	39.8	39.8	39.8
Composition of the cryoprotectant	10%(*v*/*v*) glycerol, 20%(*w*/*v*) PEG 3350	10%(*v*/*v*) glycerol, 10%(*v*/*v*) PEG 400	Reservoir solution
Drop setting	Mosquito robot	Mosquito robot	Mosquito robot
Seeding	No	No	No

**Table 3:** Data collection and processing Values in parentheses are for the outer shell.

	*Sp*CdaA, apo (PDB entry 8ofh)	*Ef*CdaA, apo (PDB entry 8ofo)	*Bs*CdaA, apo (PDB entry 8ogm)
Diffraction source	P13, PETRA III, DESY	P14, PETRA III, DESY	BL14.2, BESSY
Wavelength (Å)	0.97626	0.97624	0.91840
Temperature (K)	100	100	100
Detector	EIGER 16M	EIGER 16M	PILATUS3 2M
Crystal-to-detector distance (mm)	253.88	236	150
Rotation range per image (°)	0.1	0.1	0.1
Total rotation range (°)	360	360	200
Exposure time per image (s)	0.01	0.015	0.1
Space group	*P*2_1_	*P*6_3_22	*C*2
*a*, *b*, *c* (Å)	62.73, 102.40, 89.79	99.16, 99.16, 112.18	119.36, 39.48, 68.23
α, β, γ (°)	90, 98.33, 90	90, 90, 120	90, 95.37, 90
Mosaicity (°)	0.19	0.067	0.03
Resolution range (Å)	50.00–1.64 (1.70–1.64)	49.58–2.45 (2.52–2.45)	37.47–1.10 (1.14–1.10)
Total No. of reflections	858898 (46734)	1464104 (34616)	456821 (42671)
No. of unique reflections	130851 (9553)	12518 (884)	117680 (10937)
Completeness (%)	95.4 (73.9)	100.0 (100.0)	91.50 (85.57)†
Multiplicity	6.6 (4.9)	37.1 (39.1)	3.9 (3.9)
〈*I*/σ(*I*)〉	15.7 (0.99) [2.02]‡	17.4 (3.78)	12.35 (0.87)‡
*R*_meas_	0.06 (2.074)	0.385 (3.473)	0.04853 (1.691)
CC_1/2_ (%)	100 (39.6)	99.8 (79.2)	99.9 (60.7)
Overall *B* factor from Wilson plot (Å^2^)	28.7	59.7	15.3

† Due to crystal orientation, *C*2 space group (no kappa offset was used).

‡ The mean *I*/σ(*I*) in the outer shell is <2.0 from the resolution threshold stated in square brackets; the highest resolution limit was estimated based on CC_1/2_.

**Table 4:** Structure refinement Values in parentheses are for the outer shell.

	*Sp*CdaA, apo (PDB entry 8ofh)	*Ef*CdaA, apo (PDB entry 8ofo)	*Bs*CdaA, apo (PDB entry 8ogm)
Resolution range (Å)	48.28–1.64 (1.66–1.64)	49.58–2.45 (2.70–2.45)	37.47–1.10 (1.11–1.10)
Completeness (%)	95.37 (70.47)	99.95 (100.00)	91.5
σ Cutoff	*F* > 1.36σ(*F*)	*F* > 0.0σ(*F*)	*F* > 1.34σ(*F*)
No. of reflections, working set	130776 (3212)	12514 (3039)	117622 (10926)
No. of reflections, test set	6538 (162)	626 (152)	5912 (598)
Final *R*_cryst_	0.1920 (0.4120)	0.2096 (0.2526)	0.1713 (0.3815)
Final *R*_free_	0.2230 (0.4130)	0.2704 (0.3484)	0.2020 (0.3960)
No. of non-H atoms			
Total	7646	2296	3376
Protein	7035	2285	2319
Ligand	—	11	—
Water	611	—	1057
R.m.s. deviations			
Bond lengths (Å)	0.01	0.02	0.011
Angles (°)	0.9	1.91	1.21
Average *B* factors (Å^2^)			
Overall	41.82	60.45	
Protein	41.47	60.64	20.90
Ligand	—	21.56	—
Water	45.91	—	36.15
Ramachandran plot			
Favored regions (%)	98.54	95.56	98.67
Additionally allowed (%)	1.46	4.44	1.33
Outliers (%)	0.00	0.00	0.00
No. of monomers in the asymmetric unit/No. of dimers	6/3	2/1	2/1
